# The repertoire of Adhesion G protein-coupled receptors in adipocytes and their functional relevance

**DOI:** 10.1038/s41366-020-0570-2

**Published:** 2020-03-19

**Authors:** Tomáš Suchý, Christian Zieschang, Yulia Popkova, Isabell Kaczmarek, Juliane Weiner, Aenne-Dorothea Liebing, Mehmet Volkan Çakir, Kathrin Landgraf, Martin Gericke, John Andrew Pospisilik, Antje Körner, John T. Heiker, Dirk Dannenberger, Jürgen Schiller, Torsten Schöneberg, Ines Liebscher, Doreen Thor

**Affiliations:** 1 Rudolf Schönheimer Institute of Biochemistry, Medical Faculty, Leipzig University, Leipzig, Germany; 2 Institute for Medical Physics and Biophysics, Medical Faculty, Leipzig University, Leipzig, Germany; 3 Department of Endocrinology, Medical Faculty, Leipzig University, Leipzig, Germany; 4 Center for Pediatric Research Leipzig, Hospital for Children & Adolescents, Medical Faculty, Leipzig University, Leipzig, Germany; 5 Institute for Anatomy and Cell biology, Medical Faculty, Halle University, Halle (Saale), Germany; 6 Van Andel Research Institute, Grand Rapids, MI 49503 USA; 7 IFB Adiposity Diseases, Leipzig University, Leipzig, Germany; 8 Helmholtz Institute for Metabolic, Obesity and Vascular Research (HI-MAG) of the Helmholtz Zentrum München at the University of Leipzig and University Hospital Leipzig, Leipzig, Germany; 9 Leibniz Institute for Farm Animal Biology, Institute of Muscle Biology and Growth, Dummerstorf, Germany

**Keywords:** Obesity, Adipocytes, Fat metabolism

## Abstract

**Background:**

G protein-coupled receptors (GPCR) are well-characterized regulators of a plethora of physiological functions among them the modulation of adipogenesis and adipocyte function. The class of Adhesion GPCR (aGPCR) and their role in adipose tissue, however, is poorly studied. With respect to the demand for novel targets in obesity treatment, we present a comprehensive study on the expression and function of this enigmatic GPCR class during adipogenesis and in mature adipocytes.

**Methods:**

The expression of all aGPCR representatives was determined by reanalyzing RNA-Seq data and by performing qPCR in different mouse and human adipose tissues under low- and high-fat conditions. The impact of aGPCR expression on adipocyte differentiation and lipid accumulation was studied by siRNA-mediated knockdown of all expressed members of this receptor class. The biological characteristics and function of mature adipocytes lacking selected aGPCR were analyzed by mass spectrometry and biochemical methods (lipolysis, glucose uptake, adiponectin secretion).

**Results:**

More than ten aGPCR are significantly expressed in visceral and subcutaneous adipose tissues and several aGPCR are differentially regulated under high-caloric conditions in human and mouse. Receptor knockdown of six receptors resulted in an impaired adipogenesis indicating their expression is essential for proper adipogenesis. The altered lipid composition was studied in more detail for two representatives, ADGRG2/GPR64 and ADGRG6/GPR126. While GPR126 is mainly involved in adipocyte differentiation, GPR64 has an additional role in mature adipocytes by regulating metabolic processes.

**Conclusions:**

Adhesion GPCR are significantly involved in qualitative and quantitative adipocyte lipid accumulation and can control lipolysis. Factors driving adipocyte formation and function are governed by signaling pathways induced by aGPCR yielding these receptors potential targets for treating obesity.

## Introduction

The worldwide increasing prevalence of obesity is the number one risk factor for serious health problems such as diabetes mellitus type 2, cardiovascular disease, neurodegeneration, and nonalcoholic fatty liver [1]. Therefore, understanding the mechanisms regulating adipocyte differentiation, fat storage, and lipolysis is required for meaningful prevention and intervention of obesity.

G protein-coupled receptors (GPCR) are literally found on all cells transducing extracellular signals into intracellular responses and are involved in almost every physiological process. Due to these features GPCR represent major drug targets in medicine. Currently, about 30 to 50 percent of all clinically relevant drugs are targeted to GPCR [2, 3]. In adipose tissue, the expression of several GPCR has been demonstrated [4, 5] and their activation was linked to adipocyte function [5]. Changing cAMP levels, for example, activate the hormone sensitive lipase and the adipose triglyceride lipase leading to an increase in lipolysis [6]. So far, mainly receptors of the rhodopsin-like GPCR class have been characterized [7–12]. Expression analysis of GPCR in adipose tissue grouped 13 receptors into an ‘adipose’ cluster indicating essential functions in this tissue [4]. While most of these receptors have already been associated with adipocyte physiology, one receptor, ADGRG2/GPR64 has not yet been reported in this context. GPR64 belongs to the Adhesion GPCR (aGPCR) class. The role of aGPCR in adipose tissue is largely unknown. Besides a few reports connecting some representatives (ADGRE1/EMR1, ADGRG3/GPR97, ADGRE5/CD97) to adipose tissue inflammation [13, 14], only ADGRF5/GPR116 and ADGRG1/GPR56 have been shown to directly modulate adipogenesis and adipocyte function [15, 16].

aGPCR are an enigmatic class of GPCR characterized by their extraordinary size and modular structural composition of the N terminus. The GPCR autoproteolysis-inducing (GAIN) domain is the hallmark of this receptor class which guides autoproteolytic procession at a highly conserved cleavage motif [17]. It also marks the location of a tethered agonist sequence, referred to as the ‘Stachel’, which is necessary to induce the active conformation of most aGPCR [18–21]. Peptides derived from this sequence can be used to modify activity levels of the given receptor. Further means of activation include interaction with extracellular ligands [22–29] and mechanical forces [26, 30, 31]. The signaling pathways targeted by aGPCR are very divers. Besides the expected interaction with multiple G proteins [32, 33] and arrestins [34], activation of the Wnt pathway [35–39] and interaction with the cytoskeleton [40] have been shown. Therefore, aGPCR are of high interest in cells that undergo large dynamic changes in cell size like adipocytes, especially with regard to the known modulatory effects of mechanical stress on them [41, 42]. Yet, functional evaluation of these receptors is still in its infancy as expression, activation, and knockdown of these extraordinary large receptors are still difficult to obtain.

In this study, we investigated the expression and functional impact of the complete aGPCR class in the model cell line 3T3-L1, adipose tissue, and primary adipocytes. We evaluated the impact of receptor knockdown on adipogenesis and analyzed the effect of receptor activation on adipocyte function. This first comprehensive investigation of aGPCR in adipose tissue will guide further exploration of this receptor class with respect to their metabolic functions.

## Material and methods

### Materials

All standard chemicals were purchased from Sigma-Aldrich Chemie GmbH (Taufkrichen, Germany) and C. Roth GmbH + Co. KG (Karlsruhe, Germany). Cell culture materials and kits were obtained from ThermoFisher Scientific (Darmstadt, Germany). Primers were synthesized by Microsynth Seqlab (Göttingen, Germany) or ThermoFisher Scientific (Darmstadt, Germany). Peptide synthesis was carried out by the Core Unit Peptide Technology (Medical Faculty, Leipzig University, Germany).

### Analysis of RNA-Seq data

Publicly available RNA-Seq data (GSE76133) of adipose tissue of mice fed with chow or high-fat diet were analyzed regarding GPCR expression [43].

RNA-Seq from human samples was performed from subcutaneous adipose tissue of subjects of the Leipzig Childhood adipose cohort and analyzed towards GPCR expression in lean and overweight/obese subjects as has been previously described [44, 45]. Expression values are given as transcripts per kilobase million (TPM). To evaluate expression changes using RNAseq data of lean and obese individuals, DESeq analysis was performed.

### 3T3-L1 cell culture, differentiation, and transfection

3T3-L1 CL-173™ cells (ATCC, LGC Standards, Wesel, Germany) were cultured and differentiated as previously described [46]. For knockdown of aGPCR mRNA levels we used transient transfection with receptor-specific siRNA (sequences in Supplementary Table S1). Control siRNA did not interfere with 3T3-L1 differentiation as shown in Supplementary Fig. S1. Details are given in Supplementary Material and methods.

### Animals

Wild-type C57BL/6N mice were bred under specific pathogen-free conditions, a 12:12 h light/dark cycle, at 21 °C, and 55% humidity. Mice had free access to food and water. For diet-induced obesity, mice on a C57BL/6 background were fed a high-fat diet (60% kcal fat; ssniff Spezialdiäten, Soest, Germany) for 24 weeks, starting at 6 weeks of age [47]. Control littermates were kept on a regular chow diet (9% kcal fat; ssniff Spezialdiäten, Soest, Germany). All experiments were conducted in accordance with European Directive 2010/63/EU on the protection of animals used for scientific purposes and were performed with permission from the Animal Care and Use Committee (ACUC #T24/16, #T19/18, #TVV12/17) and the Government of the State of Saxony, Germany.

### Isolation of mouse adipocytes and stromal vascular fraction (SVF)

Mouse adipocytes and SVF were isolated from male mice sacrificed by cerebral dislocation. After preparation of periepididymal fat, the fat pads were washed in cold PBS, chopped up, and transferred into 5 ml of sterile adipocyte isolation buffer (123 mM NaCl, 5 mM KCl, 1.3 mM CaCl_2_, 5 mM glucose, 100 mM HEPES, 1% Pen/Strep, 4% BSA, and 1 mg/ml collagenase I (Worthington, Columbus, OH, USA)). To allow for collagenase digestion, fat tissue was incubated at 37 °C for 45 min shaking in a water bath (120 rpm). Undigested fat was removed by filtering (mesh size: 100 µm). After 5 min centrifugation at 1000 rpm (Megafuge 16R, ThermoFisher Scientific, Darmstadt, Germany), the supernatant containing the adipocytes was washed twice with PBS. The SVF fraction-containing cell pellet was resuspended in 2 ml erythrocytes lysis buffer (0.154 mM NH_4_Cl, 0.01 mM KHCO_3_, and 0.1 mM EDTA), incubated for 7 min at RT, centrifuged at 2000 rpm for 4 min and washed twice with PBS.

### Adipocyte staining and droplet analysis

After differentiation of 3T3-L1 fibroblasts to mature adipocytes, cells were fixed in 10% formaldehyde/PBS in two incubation steps, 5 min and 1 h, and subsequently washed with 60% isopropanol. Oil Red O (ORO) stock solution was prepared by solving 3.5 g/l ORO in isopropanol and stored at 4 °C. The working solution was prepared fresh before usage by diluting the stock solution in *d*H_2_O 60:40, incubation at RT for 20 min, and filtering (mesh size: 0.2 µm) resulting in a final concentration of 2.1 g/l ORO. For staining, fixed cells were incubated with fresh working solution of ORO for 10 min. After incubation, cells were immediately washed four times with tap water. Pictures were taken using Leica LAS EZ software (Leica Microsystems GmbH, Wetzlar, Germany) and further analyzed using ImageJ, modifying an approach described before [48]. Elution of ORO was carried out by adding 50 µl isopropanol (Carl Roth GmbH & Co. KG, Karlsruhe, Germany) and short incubation at RT while pipetting up and down. OD values were measured at 500 nm using the Sunrise™ photometer (Tecan Group Ltd., Männedorf, Switzerland).

### RNA extraction and real-time quantitative-PCR

Cells were harvested every other day after washing with PBS using BL-TG buffer and RNA isolation was performed according to manufacturer’s instructions (ReliaPrep™ RNA Miniprep Systems, Promega, Mannheim, Germany). Reverse transcription was executed using SuperScript II™ Reverse Transcriptase. For quantitative PCR, the increase of fluorescence of the SYBR green dye was measured using Platinum® SYBR-Green qPCR SuperMix-UDG, 10 ng cDNA, 1.2 µM primer mix, and a CFX Connect™ Real-Time PCR Detection System (Bio-Rad) as advised by the manufacturer. Data were normalized to beta-actin which served as recommended housekeeping gene [49]. For primer sequences see Supplementary Table S2.

### Lipid analysis

Lipid extraction was performed according to Matyash et al. [50] and HPTLC and ESI-IT MS measurements according to Engel et al. [51]. TAG fractions were independently investigated by GC analysis performed as already essentially described [52, 53]. For details see Supplementary Material and methods.

### cAMP accumulation assay

Cyclic AMP accumulation assay was performed in 96-well plates 2 days post confluence of 3T3-L1 cells. In brief, cells were washed in serum-free DMEM containing 1 mM IBMX (Sigma-Aldrich) and further incubated for 15 min in 100 µl serum-free DMEM containing 1 mM IBMX and the respective compounds. Cells were lysed using LI buffer (5 mM HEPES, 0.3% Tween-20, 0.1% BSA, and 0.5 mM IBMX). The amount of cAMP was determined using the AlphaScreen^TM^ cAMP Functional Assay (PerkinElmer, Rodgau, Germany) according to the manufacturer’s protocol using the EnVision 2105 Multimode Plate Reader (PerkinElmer, Rodgau, Germany).

### Gpr64 cell surface expression

Endogenous Gpr64 cell surface expression was determined 3 days post transfection with Gpr64-specific or control siRNA using an indirect ELISA. Thereto, cells were fixed with 4% formaldehyde for 20 min and incubated with blocking solution (media supplemented with 10% FBS) for 1 h at 37 °C. ELISA was performed using primary anti-Gpr64-antibody (2 µg/ml, AF7977, R&D Systems, Minneapolis, USA) and secondary HRP-conjugated anti-sheep-antibody (1:1,000, HAF016, R&D systems, Minneapolis, USA). Antibodies were diluted into blocking solution and incubated for 1 h at 37 °C. For visualization *o*-phenylenediamine was solved in substrate buffer (0.1 M citric acid, 0.1 M Na_2_HPO_4_) containing H_2_O_2_ and incubated for 20 min at room temperature. The reaction was stopped by adding 1 M HCl containing Na_2_SO_3_. OD values were determined at 492 nm using the Sunrise microplate reader (Tecan, Männedorf, Switzerland).

### Analyzing adipocyte function

Adiponectin secretion, glucose uptake, and lipolysis were analyzed in fully differentiated 3T3-L1 cells or mature adipocytes. For details see Supplementary Materials and methods.

### Statistical analyses

Significance was tested by either one-way or two-way ANOVA followed by indicated post hoc test or paired two-sided *t*-test as described in the figure legends. Sample size was estimated based on previous publications. *p* values < 0.05 were considered statistically significant. Statistical analyses were performed with GraphPad Prism (GraphPad Software, San Diego, California, USA).

## Results

### The majority of aGPCR is expressed in adipose tissue and differentially regulated under high-fat conditions

Publicly available RNA-Seq data from mice [43] and own human RNA-Seq data [44, 45] were analyzed regarding the GPCR expression in adipose tissue. We found 288 GPCR to be expressed in mouse adipose tissue, however, only 114 GPCR transcripts had a TPM value above 1.0. In human subcutaneous tissue, 341 GPCR were detected with 174 having a TPM value above 1.0 (Supplementary Table S3). Analyzing the class distribution of the 100 highest expressed receptors in human and mouse adipose tissues, we found that almost 75% belong to the rhodopsin class. However, about 37% of all aGPCR (12 in mouse and human tissue) are significantly expressed in adipose tissue, highlighting the importance of this class.

**Fig. 1:**
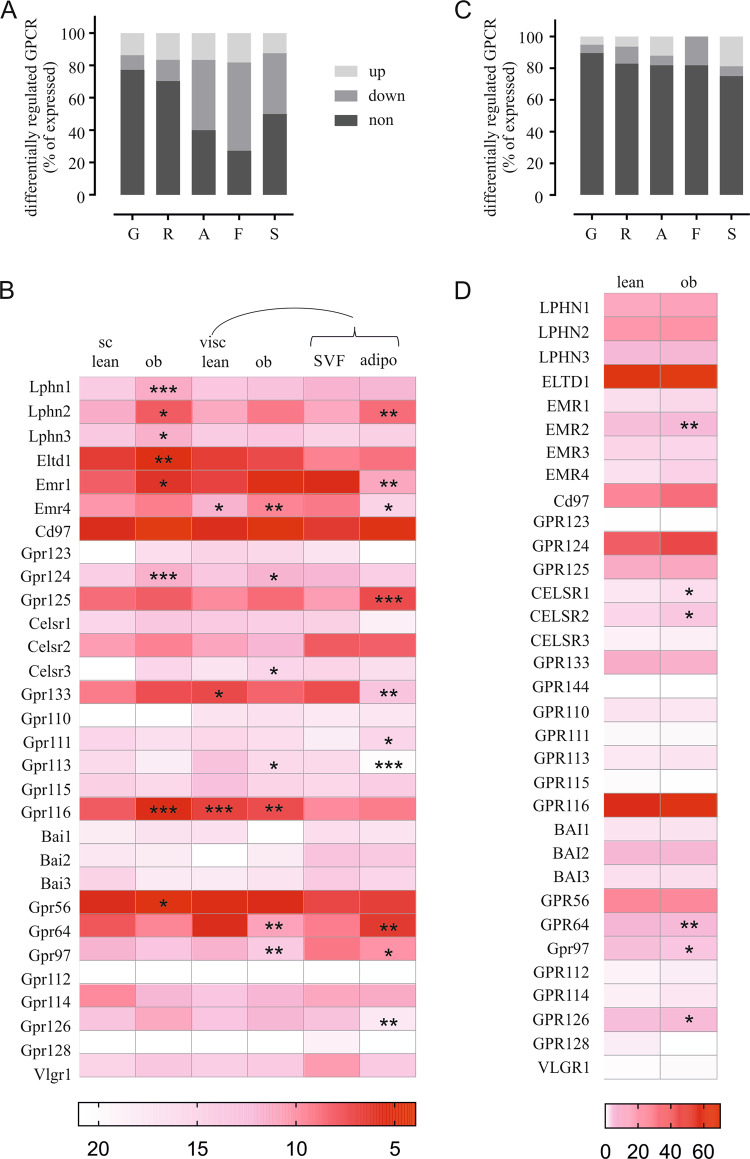
The GPCR repertoire in adipose tissue and its regulation under high-fat diet. **a** Adhesion GPCR are highly regulated under high-fat diet in mouse visceral adipose tissue. Expression changes of all detected GPCR were evaluated under high-fat diet compared with chow fed animals. Data are given as percentage of all expressed GPCR per class (GRAFS system [66]) for nonregulated (dark gray), downregulated (gray), and upregulated (light gray). **b** Heatmap showing expression of aGPCR in subcutaneous (sc) and visceral (visc) adipose tissue obtained from mice under chow and high-fat diet, as well as SVF and adipocytes isolated from visceral fat depot from lean mice. We performed qPCR analysis to evaluate the expression pattern and regulation of all 30 aGPCR in the mouse genome. Significant expression changes of subcutaneous vs. visceral adipose tissue are indicated by an asterisk in the visceral adipose tissue column. Significant changes due to high-fat diet are indicated in the high-fat diet column of each fat depot. Analogously, significant changes in SVF compared with adipocytes are marked in the adipocyte column. Given is the mean of three independent experiments each performed in triplicates. Significant changes are determined using an ordinary One-way ANOVA with Tukey’s multiple comparisons test. *p < 0.05; **p < 0.01; ***p < 0.001. **c** GPCR regulation in lean vs. obese children were obtained from RNA-Seq expression data. Significant changes were calculated using DESeq analysis. Comparable with mouse adipose tissue, Adhesion and Frizzled receptors are the most regulated GPCR. **d** Heatmap showing aGPCR expression patterns in human subcutaneous adipose tissue in lean and overweight/obese children. Analysis was performed on samples of age- and sex-matched lean [29] and overweight/obese [22] individuals. Significant aGPCR expression changes between lean and obese individuals were determined by DESeq analysis and are marked with an asterisk. *p < 0.05; **p < 0.01.

To gain insight into the potential importance of aGPCR, we compared expression changes of all GPCR classes in visceral adipose tissue between lean and obese mice. Under high-fat diet we found highest percentages of regulated receptors among Adhesion and Frizzled-type GPCR (Fig. 1a). For further details on aGPCR expression under specific diets we performed qPCR analysis in different adipose tissues and cell-fractionated visceral adipose tissue. Out of 30 aGPCR present in mice, we found 25 receptors to be expressed in subcutaneous and 28 expressed in visceral fat depots, however, 9 in either tissue only in traces (ΔCt value above 14) (Fig. 1b, Supplementary Table S4). Comparing both fat depots, we found significant differences in expression for Adgre4/Emr4, Adgrd1/Gpr133, and Gpr116. In subcutaneous fat, we mostly observed upregulation of aGPCR under high-fat conditions, out of which seven were significant. Only three receptors (Gpr64, Gpr97, Adgrg5/Gpr114) were downregulated; however, none of these reached significance (Fig. 1b). Comparing visceral fat tissue from mice fed with chow or high-fat diet [47] we found seven receptors to be significantly regulated. Emr4, Adgra2/Gpr124, and Adgrc3/Celsr3 are upregulated in obese mice, whereas Adgrf3/Gpr113, Gpr116, Gpr64, and Gpr97 were downregulated (Fig. 1b). Since fat is a heterogeneous tissue, we isolated primary adipocytes and SVF from visceral fat depot to identify the receptors specific for either fraction. Four aGPCR (Adgrl2/Lphn2, Adgra3/Gpr125, Adgrf2/Gpr111, and Gpr64) were significantly higher expressed in adipocytes, whereas Adgre1/Emr1, Emr4, Gpr133, Gpr113, Gpr97, and Adgrg6/Gpr126 were found in SVF in significantly larger amounts (Fig. 1b).

Similarly, we performed expression analysis between lean and obese human individuals. Again, Adhesion and Frizzled GPCRs classes have the highest percentage of differentially regulated receptor expression (Fig. 1c). aGPCR expression in human subcutaneous adipose tissue is comparable with the one found in mouse (Fig. 1d, Supplementary Table S4). Thus, we observed high expression of Adgrl4/Eltd1, Cd97, Gpr124, Gpr116, and Gpr56. Interestingly, we identified significant regulation of six aGPCR in lean vs. obese individuals with Gpr64 and Gpr97 being downregulated while Celsr1, Celsr2, Emr2, and Gpr126 are upregulated (Fig. 1d).

### aGPCR are dynamically expressed during differentiation of 3T3-L1 preadipocytes into adipocytes

**Fig. 2:**
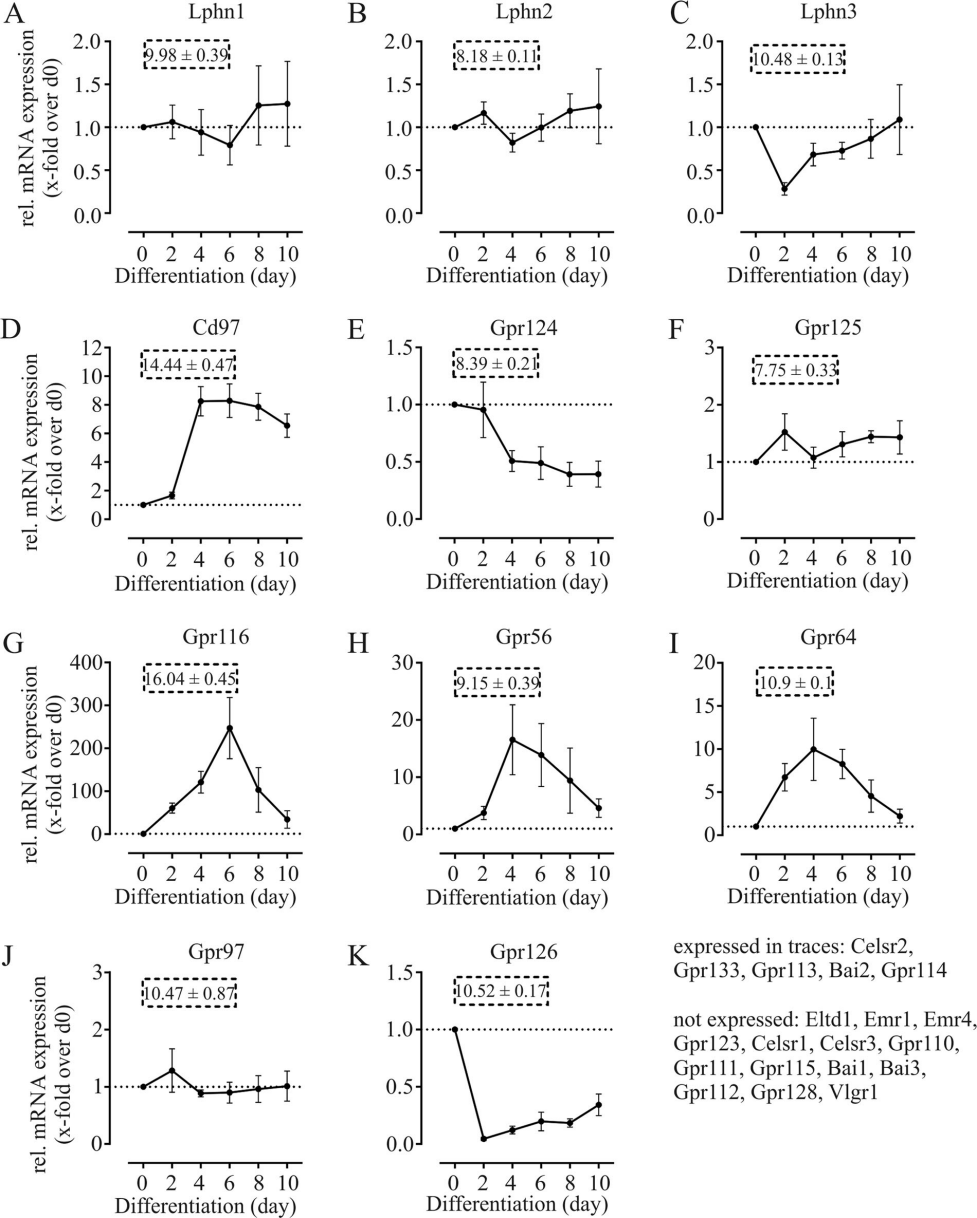
Expression of aGPCR during adipogenesis of 3T3-L1 cells. mRNA levels were determined by qPCR at six time points in the 10-day period of adipocyte differentiation and normalized to the housekeeping gene β actin (Ct = 17.23 ± 0.1). ΔCt values at day 0 are noted in dashed line box. Values of each day were further computed as relative fold change over day 0 expression and expression patterns were constructed. Raw data of qPCR measurements are noted in Supplementary Table 5. Given is the mean ± SEM (*n* = 4 biological replicates).

To investigate adipogenesis and adipocyte function, the 3T3-L1 cell line is among the most widely used cell models displaying hallmark properties of white adipose tissue including insulin-triggered glucose uptake, lipid accumulation, and lipolysis [54]. 3T3-L1 cells can be transfected and, therefore, be used to specifically study the effect of receptor knockdown on cell differentiation and function. Since each state of differentiation is characterized through varying expression of proteins, we investigated the expression of all aGPCR representatives at every other day during 3T3-L1 cell differentiation. We found 11 out of 30 aGPCR expressed at each time point investigated (Adgrl1-3/Lphn1-3, Cd97, Gpr124, Gpr125, Gpr116, Gpr56, Gpr64, Gpr97, Gpr126) (Fig. 2, Supplementary Table S5). Of those, Lphn1, Lphn2, Gpr125, and Gpr97 showed no obvious changes during differentiation (Fig. 2a, b, f, j).

We observed steady downregulation of Gpr124 (Fig. 2e) and Gpr126 (Fig. 2k) after initiating differentiation. This observation is in line with the significant expression of these receptors in SVF of adipose tissue but their absence in isolated adipocytes (Fig. 1d). Lphn3 (Fig. 2c) also shows an initial decline, in contrast to Gpr124 and Gpr126, this receptor’s expression recovers in the course of differentiation. A steady upregulation was only observed for Cd97 (Fig. 2d). Expression of Gpr116 (Fig. 2g), Gpr56 (Fig. 2h), and Gpr64 (Fig. 2i) peaks at day 4 or day 6 which confirms the expression pattern shown previously for Gpr116 [15]. All of these receptors show exceptionally high expression levels in adipose tissue and mature adipocytes (Fig. 1d). Adgrc2/Celsr2, Gpr133, Gpr113, Adgrb2/Bai2, and Gpr114 were expressed in traces throughout differentiation even though Celsr2 is expressed highly in primary adipocytes, indicating that the 3T3-L1 cell line does not represent all features of native adipocytes.

Our data reveals individual expression profiles of aGPCR in 3T3-L1 adipogenesis pointing towards specific functions of the members of this GPCR class in adipocytes.

### Knockdown of aGPCR reduces differentiation ability of 3T3-L1 cells

**Fig. 3:**
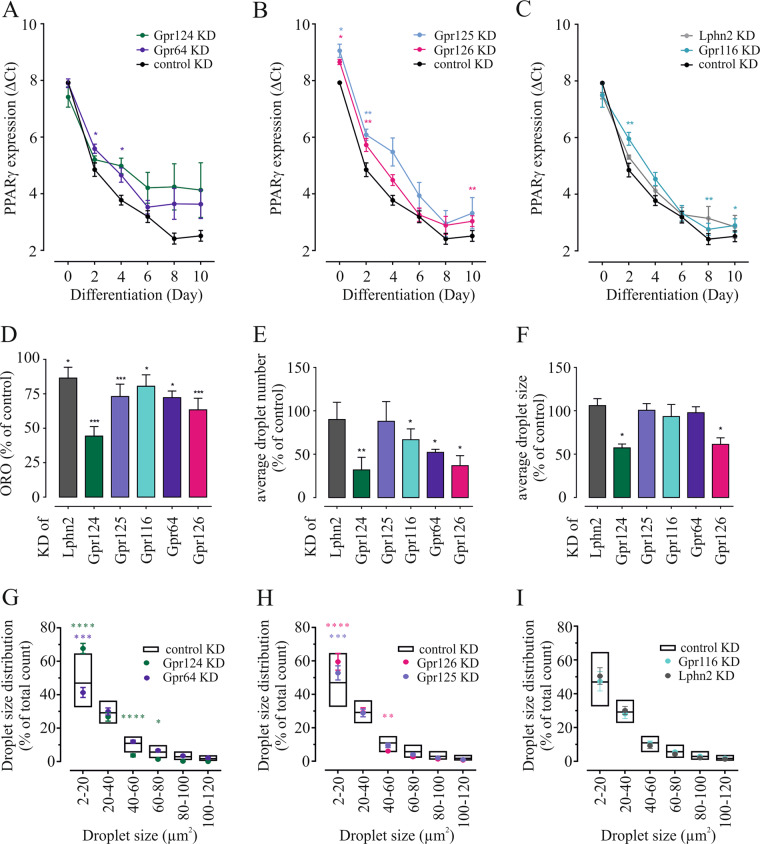
Effects of aGPCR knockdown on adipogenesis. 3T3-L1 cells were induced to differentiate under transient knockdown of the given aGPCR and compared with control-transfected cells. **a**, **b**, **c** Significant regulation of the adipogenic marker PPARγ under individual knockdown of six receptors was observed during adipogenesis. During the differentiation, we detected three different patterns of PPARγ expression (see Text for details). **d** Total lipid accumulation was measured by eluted ORO in day 10 adipocytes under receptor-specific transient knockdown and compared with control-transfected cells (Supplementary Table S7). **e** The count of lipid droplets per field of view (0.2664 mm^2^, minimum 5000 droplets counted per experiment) was lowered under knockdown of four receptors compared with control siRNA-transfected cells (Supplementary Table S7). **f** Lipid droplet size was significantly smaller under knockdown of Gpr124 and Gpr126 compared with control siRNA-transfected cells (Supplementary Table S7). **g**, **h**, **i** Analysis of lipid droplet size distribution. Size distribution of control is depicted in white bars (min to max). Given is the mean ± SEM (*n* > 3 biological replicates). Statistical significance of PPARγ expression, ORO elution and lipid droplet size and count was identified by paired *t*-test. Lipid droplet size distribution was tested by two-way ANOVA followed by Dunnett’s test for multiple comparisons. *p < 0.05; **p < 0.01; ***p < 0.001 ****p < 0.0001.

To evaluate the contribution of single aGPCR to adipogenesis, we investigated the consequences of knockdown of 10 of the 11 expressed receptors on the differentiation marker PPARγ on every other day after differentiation induction (knockdown stability shown in Supplementary Table S6). Three different patterns of PPARγ expression were identified under aGPCR knockdown. The first pattern comprises knockdown effects of Gpr124 and Gpr64, which showed no effect on initial PPARγ levels but led instead to a stop in further increase of this transcription factor starting from day 4 to day 6, respectively **(**Fig. 3a). A second group, including Gpr125 and Gpr126, showed a similar course of PPARγ expression as in wt cells, however, transcript levels were reduced from day 0 to day 10 (Fig. 3b). A third group, consisting of Lphn2 and Gpr116, displayed persistent reduction of PPARγ expression following day 2 with a wt-like course of transcription factor expression during adipogenesis (Fig. 3c). Knockdown of the remaining aGPCR tested did not show altered PPARγ expression (Supplementary Fig. S2A).

ORO staining is widely used to quantify the lipid amount in adipocytes [55]. We found that siRNA-mediated knockdown of six aGPCR (Lphn2, Gpr124, Gpr125, Gpr116, Gpr64, Gpr126) resulted in significantly reduced lipid storage (Fig. 3d, Supplementary Fig. S2B). Knockdown of Gpr124 and Gpr126 altered the average number of lipid droplets (Fig. 3e) and the average droplet size (Fig. 3f) in adipocytes. Knockdown of Gpr116 and Gpr64 caused only a reduced average droplet number (Fig. 3e, see Supplementary Table S7 for absolute values of controls). In-depth analysis of lipid droplet size distribution showed a reduced number of smaller droplets for Gpr64 knockdown, whereas knockdown of Gpr124, Gpr125, and Gpr126 have a higher number of smaller droplets (Fig. 3g/h). Knockdown of Gpr116 and Lphn2 did not result in significant alterations of droplet size distribution (Fig. 3i).

Overall, knockdown of Lphn1, Lphn3, Cd97, and Gpr56 did not significantly alter adipogenesis, whereas the reduction of Lphn2, Gpr124, Gpr125, Gpr116, Gpr64, and Gpr126 led to reduced lipid accumulation.

Evaluating the expression and the effect of receptor knockdown on PPARγ expression and droplet size we have chosen Gpr126 and Gpr64 to further investigate their impact on lipid composition and found an overall trend towards increased amounts of long chain fatty acids (Supplementary Fig. 3, Supplementary Results).

### GPR64 stimulation alters function in mature adipocytes

**Fig. 4:**
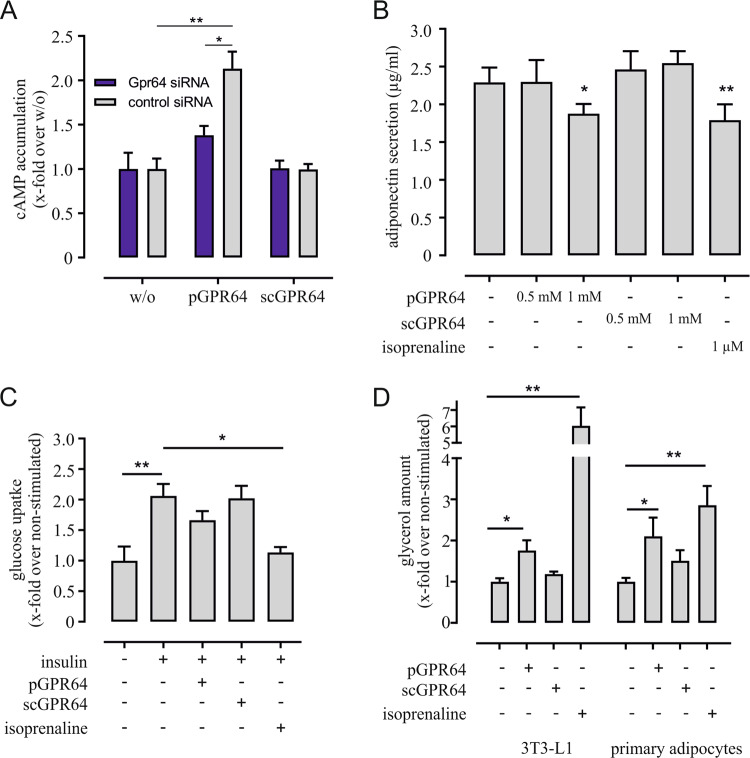
Activation of endogenous GPR64 in 3T3-L1 cells and primary adipocytes. **a** Activation of endogenous GPR64 in 3T3-L1 preadipocytes using 0.5 mM agonistic peptide increases intracellular cAMP. Receptor knockdown with siRNA specific for Gpr64 leads to a significantly reduced cAMP accumulation. A scrambled version of the Stachel-peptide (0.5 mM) does not change intracellular cAMP levels. Given is the mean ± SEM of three independent experiments each performed in triplicates (cAMP concentration: Gpr64 siRNA: 3.56 ± 0.65 nM; control siRNA: 2.83 ± 0.33 nM). **b** Stimulation of mature 3T3-L1 adipocytes with pGPR64 at given concentrations significantly decreases the amount of secreted adiponectin similar to the effect of isoprenaline. As expected, the scrambled peptide does not have an effect on adiponectin secretion. Depicted is the mean ± SEM of five to six independent experiments performed in duplicates. **c** Glucose uptake was measured in fully differentiated 3T3-L1 cells after incubation with insulin, 0.5 mM pGPR64, 0.5 mM scGPR64, and 1 µM isoprenaline. Insulin induces a significant increase in glucose uptake which is reduced by isoprenaline. The stimulating peptide pGPR64 shows a trend towards reduced insulin-induced glucose uptake, whereas the scrambled peptide does not have an effect. Given is the mean ± SEM of two (isoprenaline) to four (pGPR64, scGPR64) independent experiments. Basal glucose uptake was 2.14 ± 0.34 dpm/mg protein. **d** Stimulation of GPR64 with the agonistic peptide pGPR64 (0.5 mM) results in a significantly enhanced lipolysis in mature 3T3-L1 and primary adipocytes. As expected, control stimulation of endogenously expressed β adrenergic receptors with isoprenaline increases lipolysis, while the scrambled peptide (at same concentration as the agonistic peptide) does not alter this function. Shown is the mean ± SEM of eight independent experiments performed in triplicates (3T3-L1, basal glycerol release 27.4 ± 2.36 µg/ml) or six independent experiments done in duplicates (primary adipocytes, basal glycerol release 17.4 ± 1.59 µg/ml). Statistical significance was tested using a paired *t*-test *p < 0.05; **p < 0.01; ***p < 0.001.

GPR64 shows high expression in mature 3T3-L1 and primary adipocytes and is significantly downregulated under high-fat diet. To analyze the function of the G_s_ protein-coupled GPR64 in adipocytes we used an activating Stachel-peptide (pGPR64) and a scrambled version (scGPR64) for control purposes and tested the cells in cAMP assays [20, 56]. As expected, peptide activation of endogenously in 3T3-L1 cells expressed GPR64 induced accumulation of cAMP concentration-dependent while the scrambled peptide had no effect (Supplementary Fig. S4A). In siRNA-transfected cells with reduced cell surface expression of GPR64 (Supplementary Fig. S4B) cAMP accumulation induced by pGPR64 was significantly lower compared with control-transfected 3T3-L1 cells (Fig. 4a).

Next, we analyzed the impact of pGPR64 activation on mature 3T3-L1 adipocytes regarding adiponectin secretion, glucose uptake, and lipolysis. For control purposes, we stimulated the cells with the β-adrenergic agonist isoprenaline, which significantly reduced adiponectin secretion as expected (Fig. 4b) [57]. Similarly, we observed a significant decrease in adiponectin secretion when stimulating GPR64 with 1 mM pGPR64, whereas lower peptide concentrations and the scrambled control peptide did not have an effect (Fig. 4b). Similarly, a concentration of 0.5 mM peptide was not sufficient to result in a significant change in insulin-induced glucose uptake (Fig. 4c), however, a trend towards lower glucose resorption was observed as expected for a Gs protein-coupled receptor [58]. Further, peptide-induced activation of GPR64 significantly increased lipolysis in mature 3T3-L1 adipocytes as well as in isolated primary adipocytes (Fig. 4d). Hence, our results indicate a function of GPR64 reminiscent of other Gs protein-coupling receptors in mature adipocytes with activation of this receptor resulting in decreased adiponectin secretion and glucose uptake but increased lipolysis.

## Discussion

Based on publicly available RNA-Seq data we found a large number of aGPCR being highly expressed in mouse and human adipose tissues (Fig. 1a/c) and regulated depending on the diet (Fig. 1b/d). Our own in-depth analysis of aGPCR expression in mouse adipose tissue depots found the majority of aGPCR being present in adipose tissue. However, expression varies in quantity and with distinct preferences for visceral and subcutaneous adipose tissue for some aGPCR, while others are expressed in both (Fig. 1b). The expression pattern in mouse and human tissue is highly overlapping, indicating the importance of aGPCR in both species. Previous studies have shown that visceral fat depots have a higher impact on obesity-associated diseases compared with subcutaneous fat [59]. This could be of interest when contemplating this group of GPCR as potential targets for obesity therapy. We also find differential regulation of aGPCR expression in either subtype under high-fat diet in mice. While the majority of aGPCR is upregulated in subcutaneous adipose tissue, expression in visceral fat remains the same or even drops to lower levels (Fig. 1b). Interestingly, Gpr64 is markedly downregulated in both fat types under high-fat diet, which is also observed comparing lean and obese humans (Fig. 1d).

In the model cell line 3T3-L1 we found only a subset (11 receptors) of the 28 aGPCR identified in AT. One obvious reason for this discrepancy is that adipose tissue is much more heterogeneous in cell composition. Besides adipocytes, there are preadipocytes, endothelial cells, immune cells, stem cells, and pericytes mainly to be found in SVF [60]. It is therefore conceivable that an aGPCR preferentially expressed in the SVF cannot be found in 3T3-L1 cells as can be seen for Emr1, Emr4, Bai1, Bai3, and Vlgr1. This goes in line with the previously reported roles for Emr1, Emr4, and Bai1 as immune cell receptors [27]. This assumption cannot account for the lack of expression of Eltd1 in the model cell line as primary adipocytes show a more pronounced expression than SVF (Fig. 1b). Bai3 and Vlgr1 on the other hand have mainly been associated with neuronal expression and function, thus their role in adipose tissue remains to be elucidated.

We studied the expression pattern of all 30 murine aGPCR in 3T3-L1 cells in the course of their differentiation (Fig. 2) and investigated the effect of receptor knockdown on this process and the resulting capacity to store lipids (Fig. 3). We identified 11 aGPCR that are either continuously expressed in 3T3-L1 cells or showed down- or upregulation. Interestingly, Gpr126 and Gpr124 showed a rapidly reduced expression after adipogenesis induction (Fig. 2e/k), yet, their knockdown had the strongest effect on adipogenesis and lipid content, resulting in fewer and smaller lipid droplets and a significant decrease in PPARγ expression (Fig. 3). Both receptors seem to influence different time points in differentiation when measuring PPARγ expression. While Gpr126 knockdown results in reduced expression of this transcription factor from the beginning, Gpr124 knockdown only influences PPARγ expression after d2. These time points exactly match the downregulation of either receptor under wt conditions. It is conceivable that expression of both receptors is essential for these early steps in differentiation, yet it is similarly important to suppress these genes soon after. Neither Gpr126 nor Gpr124 have been implicated in adipogenesis before and future studies will have to unveil the specific roles of both receptors in adipogenesis. Overall, we found that knockdown of any expressed aGPCR impairs differentiation or lipid storage.

We chose 2 aGPCR for further in-depth analysis towards their contribution to adipocyte function. GPR126 and GPR64 represent potential regulators of adipogenesis induction and mature adipocyte function, respectively. Analyzing the fatty acyl compositions of TAG of fully differentiated 3T3-L1 cells, we found a trend towards longer fatty acyl chains in both, GPR126- and GPR64-knockdown cells. In addition, we observed a reduction in differentiation and lipid droplet formation, which indicates an overall reduction in lipid storage. In obesity, adipocytes display increased lipid content of mainly shorter and saturated fatty acids, which is apparently caused by impairing desaturase and elongase enzyme activity [61]. It is conceivable that the observed changes in lipid content in GPR64- and GPR126-knockdown adipocytes might represent a ‘leaner’ phenotype due to impaired differentiation. However, a link between GPCR signal transduction and lipid composition has not been established yet.

Expression analysis of Gpr64 (Fig. 2i) and the observed effects of siRNA-mediated knockdown on PPARγ levels (Fig. 3a) suggest only a minor impact of this receptor on adipogenesis. Based on its strong expression in primary and mature 3T3-L1 adipocytes (Figs. 1b and 2i) as well as its regulation during obesity (Fig. 1b/d), a role of GPR64 in mature adipocyte function is conceivable. Indeed, activation of the receptor by the tethered peptide agonist [20], modulated adipocyte-specific features like hormone secretion (Fig. 4b), and lipolysis (Fig. 4d). These effects have been described for other receptors raising intracellular cAMP levels like β adrenergic receptors [62, 63]. As obesity has been associated with increased basal lipolysis [64] and decreases in adiponectin levels [65] resulting in increased tissue inflammation or insulin resistance, downregulation of GPR64 in obese individuals could be interpreted as protective mechanism to reduce the burden in overweight conditions.

In summary, we present a comprehensive picture of aGPCR expression and the effect of their knockdown during adipogenesis. Further, we identified Gpr126 to be essential for the development of mature adipocytes and we demonstrated that other members of the aGPCR class, exemplarily shown for GPR64, are necessary for the modulation of mature adipocyte function.

## Supplementary information

Supplemental Information

Suppl. Table S1

Suppl. Table S2

Suppl. Table S3

Suppl. Table S4

Suppl. Table S5

Suppl. Table S6

Suppl. Table S7

Suppl. Table S8

## References

[1] Qatanani M, Lazar MA (2007). Mechanisms of obesity-associated insulin resistance: many choices on the menu. Genes Dev.

[2] Insel PA, Sriram K, Wiley SZ, Wilderman A, Katakia T, McCann T (2018). GPCRomics: GPCR expression in cancer cells and tumors identifies new, potential biomarkers and therapeutic targets. Front Pharmacol.

[3] Hauser AS, Attwood MM, Rask-Andersen M, Schiöth HB, Gloriam DE (2017). Trends in GPCR drug discovery: new agents, targets and indications. Nat Rev Drug Discov.

[4] Regard JB, Sato IT, Coughlin SR (2008). Anatomical profiling of G protein-coupled receptor expression. Cell.

[5] Amisten S, Neville M, Hawkes R, Persaud SJ, Karpe F, Salehi A (2015). An atlas of G-protein coupled receptor expression and function in human subcutaneous adipose tissue. Pharmacol Ther.

[6] Carmen G-Y, Víctor S-M (2006). Signalling mechanisms regulating lipolysis. Cell Signal.

[7] Gnad T, Scheibler S, Kügelgen I, von, Scheele C, Kilić A, Glöde A (2014). Adenosine activates brown adipose tissue and recruits beige adipocytes via A2A receptors. Nature.

[8] Hong Y-H, Nishimura Y, Hishikawa D, Tsuzuki H, Miyahara H, Gotoh C (2005). Acetate and propionate short chain fatty acids stimulate adipogenesis via GPCR43. Endocrinology.

[9] Li H, Fong C, Chen Y, Cai G, Yang M (2010). Beta-adrenergic signals regulate adipogenesis of mouse mesenchymal stem cells via cAMP/PKA pathway. Mol Cell Endocrinol.

[10] Miller CN, Yang J-Y, England E, Yin A, Baile CA, Rayalam S (2015). Isoproterenol increases uncoupling, glycolysis, and markers of beiging in mature 3T3-L1 adipocytes. PLoS ONE.

[11] Ahmed K, Tunaru S, Tang C, Müller M, Gille A, Sassmann A (2010). An autocrine lactate loop mediates insulin-dependent inhibition of lipolysis through GPR81. Cell Metab.

[12] Cai T-Q, Ren N, Jin L, Cheng K, Kash S, Chen R (2008). Role of GPR81 in lactate-mediated reduction of adipose lipolysis. Biochem Biophys Res Commun.

[13] Kolehmainen M, Ulven SM, Paananen J, Mello V, de, Schwab U, Carlberg C (2015). Healthy Nordic diet downregulates the expression of genes involved in inflammation in subcutaneous adipose tissue in individuals with features of the metabolic syndrome. Am J Clin Nutr.

[14] Shi J, Zhang X, Wang S, Wang J, Du B, Wang Z (2016). Gpr97 is dispensable for metabolic syndrome but is involved in macrophage inflammation in high-fat diet-induced obesity in mice. Sci Rep.

[15] Nie T, Hui X, Gao X, Li K, Lin W, Xiang X (2012). Adipose tissue deletion of Gpr116 impairs insulin sensitivity through modulation of adipose function. FEBS Lett.

[16] Al Hasan M, Roy P, Dolan S, Martin PE, Patterson S, Bartholomew C (2019). Adhesion G-protein coupled receptor 56 is required for 3T3-L1 adipogenesis. J Cell Physiol.

[17] Araç D, Boucard AA, Bolliger MF, Nguyen J, Soltis SM, Südhof TC (2012). A novel evolutionarily conserved domain of cell-adhesion GPCRs mediates autoproteolysis. EMBO J.

[18] Liebscher I, Schön J, Petersen SC, Fischer L, Auerbach N, Demberg LM (2014). A tethered agonist within the ectodomain activates the adhesion G protein-coupled receptors GPR126 and GPR133. Cell Rep.

[19] Stoveken HM, Hajduczok AG, Xu L, Tall GG (2015). Adhesion G protein-coupled receptors are activated by exposure of a cryptic tethered agonist. Proc Natl Acad Sci USA.

[20] Demberg LM, Rothemund S, Schöneberg T, Liebscher I (2015). Identification of the tethered peptide agonist of the adhesion G protein-coupled receptor GPR64/ADGRG2. Biochem Biophys Res Commun.

[21] Müller A, Winkler J, Fiedler F, Sastradihardja T, Binder C, Schnabel R (2015). Oriented cell division in the C. elegans embryo is coordinated by G-protein signaling dependent on the adhesion GPCR LAT-1. PLoS Genet.

[22] Luo R, Jeong S-J, Yang A, Wen M, Saslowsky DE, Lencer WI (2014). Mechanism for adhesion G protein-coupled receptor GPR56-mediated RhoA activation induced by collagen III stimulation. PLoS ONE.

[23] Paavola KJ, Sidik H, Zuchero JB, Eckart M, Talbot WS (2014). Type IV collagen is an activating ligand for the adhesion G protein-coupled receptor GPR126. Sci Signal.

[24] Stacey M, Chang G-W, Davies JQ, Kwakkenbos MJ, Sanderson RD, Hamann J (2003). The epidermal growth factor-like domains of the human EMR2 receptor mediate cell attachment through chondroitin sulfate glycosaminoglycans. Blood.

[25] Kwakkenbos MJ, Pouwels W, Matmati M, Stacey M, Lin H-H, Gordon S (2005). Expression of the largest CD97 and EMR2 isoforms on leukocytes facilitates a specific interaction with chondroitin sulfate on B cells. J Leukoc Biol.

[26] Petersen SC, Luo R, Liebscher I, Giera S, Jeong S-J, Mogha A (2015). The adhesion GPCR GPR126 has distinct, domain-dependent functions in Schwann cell development mediated by interaction with laminin-211. Neuron.

[27] Hamann J, Hsiao C-C, Lee CS, Ravichandran KS, Lin H-H (2016). Adhesion GPCRs as modulators of immune cell function. Handb Exp Pharmacol.

[28] Wang T, Ward Y, Tian L, Lake R, Guedez L, Stetler-Stevenson WG (2005). CD97, an adhesion receptor on inflammatory cells, stimulates angiogenesis through binding integrin counterreceptors on endothelial cells. Blood.

[29] Hamann J, Vogel B, van Schijndel GM, van Lier RA (1996). The seven-span transmembrane receptor CD97 has a cellular ligand (CD55, DAF). J Exp Med.

[30] Scholz N, Gehring J, Guan C, Ljaschenko D, Fischer R, Lakshmanan V (2015). The adhesion GPCR latrophilin/CIRL shapes mechanosensation. Cell Rep.

[31] Wilde C, Fischer L, Lede V, Kirchberger J, Rothemund S, Schöneberg T (2016). The constitutive activity of the adhesion GPCR GPR114/ADGRG5 is mediated by its tethered agonist. FASEB J.

[32] Paavola KJ, Hall RA (2012). Adhesion G protein-coupled receptors: signaling, pharmacology, and mechanisms of activation. Mol Pharmacol.

[33] Liebscher I, Schoneberg T (2016). Tethered agonism: a common activation mechanism of adhesion GPCRs. Handb Exp Pharmacol.

[34] Stephenson JR, Paavola KJ, Schaefer SA, Kaur B, van Meir EG, Hall RA (2013). Brain-specific angiogenesis inhibitor-1 signaling, regulation, and enrichment in the postsynaptic density. J Biol Chem.

[35] Zhou Y, Nathans J (2014). Gpr124 controls CNS angiogenesis and blood–brain barrier integrity by promoting ligand-specific canonical wnt signaling. Dev Cell.

[36] Vanhollebeke B, Stone OA, Bostaille N, Cho C, Zhou Y, Maquet E (2015). Tip cell-specific requirement for an atypical Gpr124- and Reck-dependent Wnt/β-catenin pathway during brain angiogenesis. Elife.

[37] Posokhova E, Shukla A, Seaman S, Volate S, Hilton MB, Wu B (2015). GPR124 functions as a WNT7-specific coactivator of canonical β-catenin signaling. Cell Rep.

[38] Li X, Roszko I, Sepich DS, Ni M, Hamm HE, Marlow FL (2013). Gpr125 modulates Dishevelled distribution and planar cell polarity signaling. Development.

[39] Wu Y, Chen W, Gong L, Ke C, Wang H, Cai Y (2018). Elevated G-protein receptor 125 (GPR125) expression predicts good outcomes in colorectal cancer and inhibits Wnt/β-catenin signaling pathway. Med Sci Monit.

[40] Hilbig D, Sittig D, Hoffmann F, Rothemund S, Warmt E, Quaas M (2018). Mechano-dependent phosphorylation of the PDZ-binding motif of CD97/ADGRE5 modulates cellular detachment. Cell Rep.

[41] Shoham N, Gefen A (2012). The influence of mechanical stretching on mitosis, growth, and adipose conversion in adipocyte cultures. Biomech Model Mechanobiol.

[42] Li R, Liang L, Dou Y, Huang Z, Mo H, Wang Y (2015). Mechanical stretch inhibits mesenchymal stem cell adipogenic differentiation through TGFβ1/Smad2 signaling. J Biomech.

[43] Vernia S, Edwards YJ, Han MS, Cavanagh-Kyros J, Barrett T, Kim JK (2016). An alternative splicing program promotes adipose tissue thermogenesis. Elife.

[44] Landgraf K, Rockstroh D, Wagner IV, Weise S, Tauscher R, Schwartze JT (2015). Evidence of early alterations in adipose tissue biology and function and its association with obesity-related inflammation and insulin resistance in children. Diabetes.

[45] Dalgaard K, Landgraf K, Heyne S, Lempradl A, Longinotto J, Gossens K (2016). Trim28 haploinsufficiency triggers Bi-stable epigenetic obesity. Cell.

[46] Buerger F, Müller S, Ney N, Weiner J, Heiker JT, Kallendrusch S (2017). Depletion of Jmjd1c impairs adipogenesis in murine 3T3-L1 cells. Biochim Biophys Acta Mol Basis Dis..

[47] Braune J, Weyer U, Hobusch C, Mauer J, Brüning JC, Bechmann I (2017). IL-6 regulates M2 polarization and local proliferation of adipose tissue macrophages in obesity. J Immunol.

[48] Deutsch MJ, Schriever SC, Roscher AA, Ensenauer R (2014). Digital image analysis approach for lipid droplet size quantitation of oil red O-stained cultured cells. Anal Biochem.

[49] Zhang J, Tang H, Zhang Y, Deng R, Shao L, Liu Y (2014). Identification of suitable reference genes for quantitative RT-PCR during 3T3-L1 adipocyte differentiation. Int J Mol Med.

[50] Matyash V, Liebisch G, Kurzchalia TV, Shevchenko A, Schwudke D (2008). Lipid extraction by methyl-tert-butyl ether for high-throughput lipidomics. J Lipid Res.

[51] Engel KM, Schiller J, Müller K, Dannenberger D, Jakop U (2017). The phospholipid composition of kangaroo spermatozoa verified by mass spectrometric lipid analysis. Lipids.

[52] Popkova Y, Meusel A, Breitfeld J, Schleinitz D, Hirrlinger J, Dannenberger D (2015). Nutrition-dependent changes of mouse adipose tissue compositions monitored by NMR, MS, and chromatographic methods. Anal Bioanal Chem.

[53] Dannenberger D, Nuernberg G, Nuernberg K, Will K, Schauer N, Schmicke M (2017). Effects of diets supplemented with n–3 or n–6 PUFA on pig muscle lipid metabolites measured by non-targeted LC–MS lipidomic profiling. Journal of Food Composition and Analysis.

[54] Ruiz-Ojeda FJ, Rupérez AI, Gomez-Llorente C, Gil A, Aguilera CM (2016). Cell models and their application for studying adipogenic differentiation in relation to obesity: a review. Int J Mol. Sci.

[55] Ramírez-Zacarías JL, Castro-Muñozledo F, Kuri-Harcuch W (1992). Quantitation of adipose conversion and triglycerides by staining intracytoplasmic lipids with oil red O. Histochemistry.

[56] Balenga N, Azimzadeh P, Hogue JA, Staats PN, Shi Y, Koh J (2017). Orphan adhesion GPCR GPR64/ADGRG2 is overexpressed in parathyroid tumors and attenuates calcium-sensing receptor-mediated signaling. J Bone Miner Res.

[57] Cong L, Chen K, Li J, Gao P, Li Q, Mi S (2007). Regulation of adiponectin and leptin secretion and expression by insulin through a PI3K-PDE3B dependent mechanism in rat primary adipocytes. Biochem J.

[58] Kashiwagi A, Huecksteadt TP, Foley JE (1983). The regulation of glucose transport by cAMP stimulators via three different mechanisms in rat and human adipocytes. J Biol Chem.

[59] Fox CS, Massaro JM, Hoffmann U, Pou KM, Maurovich-Horvat P, Liu C-Y (2007). Abdominal visceral and subcutaneous adipose tissue compartments: association with metabolic risk factors in the Framingham Heart Study. Circulation.

[60] Han S, Sun HM, Hwang K-C, Kim S-W (2015). Adipose-derived stromal vascular fraction cells: update on clinical utility and efficacy. Crit Rev Eukaryot Gene Expr.

[61] Vessby B (2003). Dietary fat, fatty acid composition in plasma and the metabolic syndrome. Curr Opin Lipidol.

[62] Collins S (2011). β-adrenoceptor signaling networks in adipocytes for recruiting stored fat and energy expenditure. Front Endocrinol.

[63] Delporte M-L, Funahashi T, Takahashi M, Matsuzawa Y, Brichard SM (2002). Pre- and post-translational negative effect of beta-adrenoceptor agonists on adiponectin secretion: in vitro and in vivo studies. Biochem J.

[64] Reynisdottir S, Langin D, Carlström K, Holm C, Rössner S, Arner P (1995). Effects of weight reduction on the regulation of lipolysis in adipocytes of women with upper-body obesity. Clin Sci.

[65] Arita Y, Kihara S, Ouchi N, Takahashi M, Maeda K, Miyagawa J (1999). Paradoxical decrease of an adipose-specific protein, adiponectin, in obesity. Biochem Biophys Res Commun.

[66] Schiöth HB, Fredriksson R (2005). The GRAFS classification system of G-protein coupled receptors in comparative perspective. Gen Comp Endocrinol.

