# Supplementary material for: The repertoire of Adhesion G protein-coupled receptors in adipocytes and their functional relevance
Source: Int J Obes (Lond). 2020 Mar 19;44(10):2124–36. doi: 10.1038/s41366-020-0570-2 (PMC7508673; doi:10.1038/s41366-020-0570-2)
Supplement: Supplementary file 1 — Supplemental Information [file 41366_2020_570_MOESM1_ESM.pdf]

Supplemental Information

Supplemental Material and Methods

3T3-L1 cell culture, differentiation, and transfection

3T3-L1 cells were cultured in CELLSTAR® Filter Cap Cell Culture Flasks (GBO, Frickenhausen, Germany) in culture medium (DMEM supplemented with 10 % FBS, 100 U/ml penicillin and 100 µg/ml streptomycin) at 37 °C in a humidified atmosphere (5 % CO_2). Splitting of cells was carried out in a way, that cells never become confluent with a seeding density of 125,000 cells to 250,000 cells per T175 flask.

For differentiation into mature adipocytes, 3T3-L1 cells were grown in culture medium until confluence. Two days post reaching confluence, culture medium was replaced by differentiation medium (culture medium containing 1 µg/ml insulin, 0.25 µM dexamethasone, 0.5 mM IBMX, 2 µM rosiglitazone). After 3 days, the media was changed to culture medium containing 1 µg/ml insulin for further 3 days. Afterwards, cell culture medium was changed every 2 days. On day 10, differentiation to mature adipocytes was complete and cells were processed for analysis.

To reduce the mRNA levels of aGPCR we used an siRNA mediated knock-down approach. Oligos of siRNA specifically targeting mouse aGPCR were designed and provided by Biocat (Heidelberg, Germany) or ThermoFisher Scientific (Darmstadt, Germany) including non-coding control siRNA (for sequences see **Suppl. Tab. S1**). After reconstitution of siRNA in reconstitution buffer to 5 µM concentration, 0.216 µl siRNA was pre-incubated with 17.89 µl Opti-MEM and 0.18 µl RNAiMAX per well (96-well plate) for 20 minutes at 37 °C in a humidified atmosphere (5 % CO_2). 10,000 cells were added in 90 µl DMEM and 100 µl DMEM were added the following day. Day 4 after seeding was set as day 0 of differentiation.

Lipid extraction

Samples were transferred to 1 ml methanol containing 1 mM *t*-butylhydroxytoluene (BHT) preventing oxidation in ball mill tubes (Precellys® ceramic-kit, CKMIX, Bertin GmbH, Frankfurt am Main, Germany) and homogenized using the tissue homogenizer Precellys® 24 (VWR, Radnor, Pennsylvania, USA) for 2 × 15 sec at 5,000 rpm, and put on ice immediately. After transfer of the homogenized samples into glass vials, ball mill tubes were washed twice with 1 ml methyl-*tert*-butyl ether (MTBE) and a total volume of 4 ml MTBE was added to the samples. Samples were incubated for 1 h at 4 °C and 800 rpm. To achieve phase separation, 1 ml dH_2O was added to each sample followed by 10 minutes of centrifugation at 4 °C and 3,500 rpm. To maximize the extraction yields, the pellet was subjected to a second extraction step with 2 ml MTBE and 0.5 ml dH_2O. The obtained organic phases were combined MTBE was removed by evaporation under a gentle nitrogen steam. The samples were stored at -20 °C until further experiments.

High-Performance Thin-Layer Chromatography Electrospray ionization ion trap mass spectrometry (HPTLC ESI-IT MS)

Lipid extracts were dissolved to give 5 µg/µl solutions in chloroform and 10 µl of each sample were sprayed onto an HPTLC silica gel 60 plate (Merck KGaA, Darmstadt, Germany) using a CAMAG® Linomat 5 semi-automatic sample application system (CAMAG, Berlin, Germany). Plates were developed in vertical TLC chambers with hexane-diethyl ether-acetic acid (80:20:1, by vol) as the solvent system. Lipids were visualized by spraying the entire plate with primuline (Direct Yellow 59, Sigma-Aldrich, Taufkirchen, Germany). Upon illumination with UV light (366 nm), triacylglycerols (TAG) were detected as colored spots. These were pencil-marked and automatically eluted by a Plate Express™ TLC plate reader (Advion, Ithaca, NY, USA) with methanol as solvent and subsequently directly infused into the ESI mass spectrometer.

ESI-IT (electrospray ionization) MS was performed on an Amazon SL mass spectrometer (Bruker Daltonics GmbH, Bremen, Germany) by using direct infusion. The following conditions were used: spray voltage 4.5 kV, end plate offset 500 V, nebulizer gas 7.3 psi, drying gas (N_2) 3 l/min, capillary temperature 180 °C, flow rate 10 µl/min, sheath gas (He) flow rate 25 a.u.. The spectra were recorded in the positive ion mode with enhanced resolution. For data acquisition and subsequent analysis, the Bruker Trap Control and Data Analysis version 4.1 software (Bruker Daltonics GmbH, Bremen, Germany) were used, respectively. Assignments of all observed *m/z* ratios are summarized in **Suppl. Tab. S8**.

Gas Chromatography with Flame-Ionization Detection (GC-FID)

All solvents contained 0.005 % (w/v) of BHT to prevent the oxidation of polyunsaturated fatty acids (PUFAs). The lipid extracts (vide supra) were re-dissolved in 300 µl of toluene, and an aliquot was used for methyl ester preparation. Next, 2 ml of 0.5 M sodium methoxide in methanol was added to the samples, which were shaken in a 60 °C water bath for 10 min. Subsequently, 1 ml of 14 % boron trifluoride in methanol was added to the mixture, which was then shaken for an additional 10 min at 60 °C. Saturated $NaHCO_3$ solution (2 ml) was added, and the fatty acid methyl esters (FAMEs) were extracted 3 times in 2 ml of *n*-hexane. The solvent containing the FAMEs was reduced to dryness and the FAMEs were resuspended in 100 µl of n-hexane and stored at −18 °C until analysis. The detailed fatty acid analysis was performed using capillary GC with a CP-Sil 88 CB column (100 m × 0.25 mm, Agilent, Santa Clara, CA, USA) that was installed in a Perkin Elmer gas chromatograph Clarus 680 with a flame ionization detector and split injection (Perkin Elmer Instruments, Shelton, CT, USA). The detailed GC conditions were the same as previously described. The initial oven temperature was 150 °C, which was held for 5 min; subsequently, the temperature was increased to 175 °C and then to 200 °C at a rate of 2 °C min^{-1} and held constant for 10 min. Finally, the temperature was increased to 225 °C at a rate of 1.5 °C min^{-1} and held constant for 25 min. Hydrogen was used as the carrier gas at a flow rate of 1 ml min^{-1}. The reference

standard FAME Mix was obtained from Sigma-Aldrich (Deisenhofen, Germany). Additionally, individual methyl esters of 18:4n-3, 22:4n-6, and 22:5n-3 were purchased from Matreya (Pleasant Gap, PA, USA). Methyl esters of 18:1*trans*-11 and 18:1*cis*-11 were purchased from Larodan Fine Chemicals (Malmö, Sweden). The fatty acid profile was calculated by the internal standard method and 19:0 was used as the internal standard. The method was calibrated using 5 different concentrations ranging from 18 to 376 ng/µl for each investigated fatty acid.

Adiponectin secretion

Adiponectin secretion was analyzed on fully differentiated 3T3-L1 cells starved in FBS-free media overnight. Secretion rate was obtained measuring total adiponectin amount in the media accumulated within 24 h with stimulation of the indicated peptides and controls using a mouse adiponectin mouse ELISA kit (ThermoFisher Scientific, Darmstadt, Germany) following the manufacturer's instructions.

Glucose uptake

Glucose uptake was measured in 12-well plates on fully differentiated 3T3-L1 using [^{3}H]-labeled deoxy-D-glucose (Perkin Elmer, Rodgau, Germany). Cells were starved in FBS-free media overnight and then incubated with indicated substances in low glucose (5.6 mM), serum-free media for 1 h. Glucose uptake was induced by adding 100 nM insulin for 15 min prior to addition of 0.5 µCi/ml [^{3}H]-deoxy-D-glucose, 100 µM 2-deoxy-glucose for 30 min. Media was removed and the cells were washed twice with ice-cold PBS and lysed by RIPA-buffer. Glucose uptake was determined from cell lysate using a scintillation counter (PerkinElmer, Rodgau, Germany) and normalized to protein content analyzed using the Pierce BCA kit (ThermoFisher Scientific, Darmstadt, Germany).

Determination of lipolysis

Lipolysis rate was determined in fully differentiated 3T3-L1 cells and primary mouse adipocytes measuring free glycerol. 3T3-L1 cells were starved for 5 h in DMEM containing 5.6 mM glucose and 1 % FBS. Respective compounds were added for further incubation for 16 h. To determine lipolysis in primary mouse adipocytes, 30 µl freshly isolated adipocytes were added to 100 µl serum free medium containing the indicated compounds. The amount of glycerol was determined using Free Glycerol Reagent (Sigma-Aldrich, Taufkirchen, Germany). 150 µl of Free Glycerol Reagent were added to 20 µl of supernatant and incubated for 10 min at 37 °C and OD values measured at 540 nm using the Sunrise™ photometer (Tecan Group Ltd., Männedorf, Switzerland).

Supplemental Results

Knock-down of Gpr64 and Gpr126 alters TAG composition of 3T3-L1 cells

Evaluating the expression (**Fig. 2**) and the effect of receptor knock-down on PPARγ expression and droplet size (**Fig. 3**) we have chosen Gpr126 and Gpr64 to investigate their function in 3T3-L1 cells in more detail as they represent regulators of adipocyte differentiation and mature adipocyte function, respectively. To investigate the influence of Gpr64 and Gpr126 knock-down on the composition of storage lipids in 3T3-L1 cells, analysis of HPTLC-separated TAG fractions was performed by ESI-MS. In line with the significant reduction in droplet number and droplet size, we observed significantly reduced free fatty acids under Gpr126 knock-down conditions (**Suppl. Fig. S3A**). The most prominent peaks, assigned in both control samples, are TAG 48:2 (*m/z* 825.6), TAG 47:1 (*m/z* 813.6), TAG 46:1 (*m/z* 799.6), and TAG 49:2 (*m/z* 839.6) followed by TAG 48:1 (*m/z* 827.6) in the case of Gpr64 (**Suppl. Fig. S3D**) and TAG 47:2 (*m/z* 811.6) in the case of Gpr126 (**Suppl. Fig. S3E**). Assignments of all observed *m/z* ratios are summarized in **Suppl. Tab. S8**.

To achieve in-depth information about the fatty acyl compositions of TAG, gas chromatographic analysis (GC) was performed. In general, palmitic acid (C16:0) was the most common saturated fatty acid, followed by myristic (14:0) and heptadecanoic (17:0) acid. Stearic acid (18:0), however, was much more abundant in Gpr64 (**Suppl. Fig. S3B**) and Gpr126 knock-down cells (**Suppl. Fig. S3C**). The 2 most ample mono-unsaturated fatty acids were palmitoleic acid (16:1) and oleic acid (18:1*cis*-9). Comparing the values for the control and the Gpr64 as well as Gpr126 knock-down samples we noted a significant increase in the amount of the saturated acids, palmitic acid (C16:0) as well as heptadecanoic (C17:0) and stearic (18:0) acid, respectively. This was accompanied by a reduction of the unsaturated fatty acids, myristoleic (14:1*cis*-9) and palmitoleic (C16:1*cis*-9) acids. An exception from this pattern was the increase of the unsaturated oleic acids (C18:1*cis*-9 and 11) and poly-unsaturated fatty acids (PUFA) in general.

Supplemental Figures and Figure legends

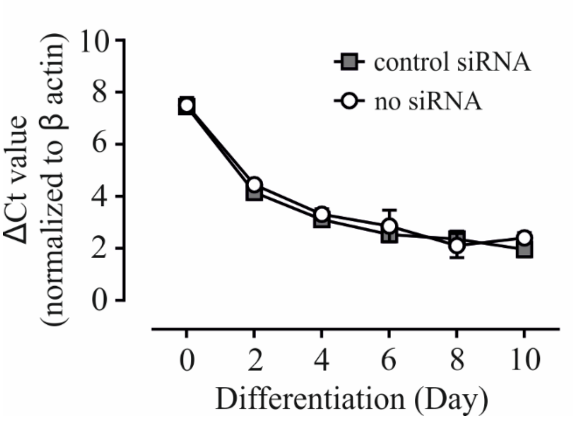

Suppl. Fig. S1. The effect of control siRNA transfection on 3T3-L1 differentiation. PPARγ expression as marker for 3T3-L1 differentiation was monitored in siRNA-transfected and non-transfected cells. No differences were observed between no transfection and control siRNA transfection. Given is the mean ± SEM of ΔCt values normalized to β actin serving as a housekeeping gene of 4 independent experiments each performed in triplicates.

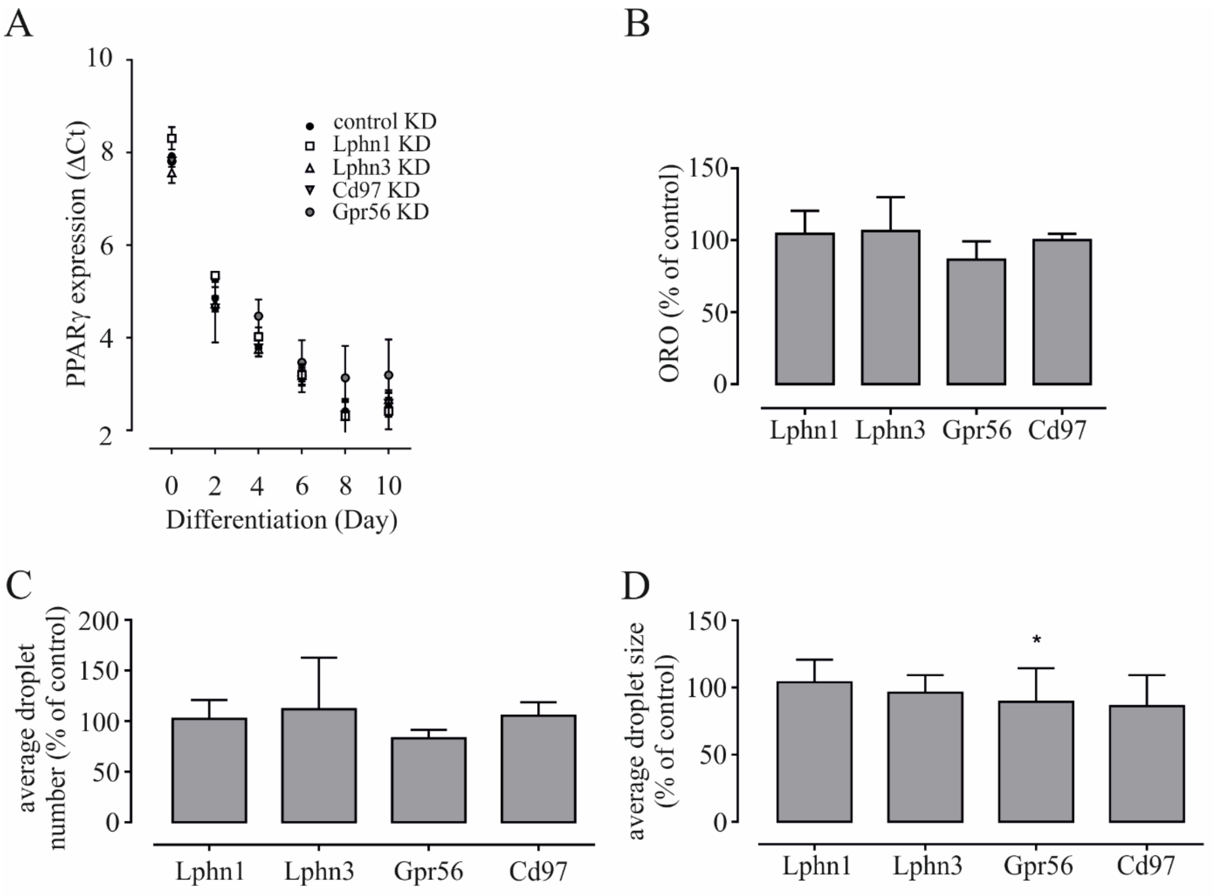

E

droplet size [µm^2]	Lphn1	Lphn3	Cd97	Gpr56	Control
0-20	48.04 ± 6.83	51.25 ± 5.15	62.13 ± 2.59*	40.96 ± 2.94*	48.28 ± 1.84
20-40	27.86 ± 3.26	27.68 ± 3.74	26.93 ± 0.51	35.62 ± 0.65*	29.21 ± 0.82
40-60	11.74 ± 2.14	11.36 ± 1.51	5.21 ± 0.74*	12.44 ± 0.94	10.34 ± 0.60
60-80	5.92 ± 1.27	5.05 ± 1.04	2.43 ± 0.69	5.24 ± 0.64	5.32 ± 0.42
80-100	2.98 ± 0.67	2.29 ± 0.36	1.48 ± 0.69	2.89 ± 0.39	2.72 ± 0.26
100-120	1.56 ± 0.49	1.20 ± 0.15	0.85 ± 0.42	1.33 ± 0.24	1.64 ± 0.16

Suppl. Fig. S2. Effects of aGPCR knock-down on adipogenesis. Analysis of the effect of transient knock-down of the highly expressed aGPCR Lphn1, Lphn3, Gpr56, and Cd97 in 3T3-L1 cells did not yield significant changes in PPARγ expression A), oil red O staining (B) or lipid droplet number (C). Size of lipid droplets was significantly lower in case of Gpr56 knock-down (D) when compared to control transfected cells (**Suppl. Tab. S7**). (E) In-depth analysis of lipid droplet size distribution displayed significant reduction in smaller droplets for Gpr56 knock-down and an increased number of small droplets in Cd97 knock-down. Given is the mean ± SEM (n > 3 biological replicates). Statistical significance of PPARγ expression, ORO elution and lipid droplets count and size was identified by paired t-test. Lipid droplet size distribution was tested by 2-way ANOVA followed by Dunnett's test for multiple comparisons. *$p < 0.05$. Please note, we were not successful in identifying an siRNA that sufficiently induced knock-down of Gpr97 even though we tested several commercially available siRNAs from different distributers.

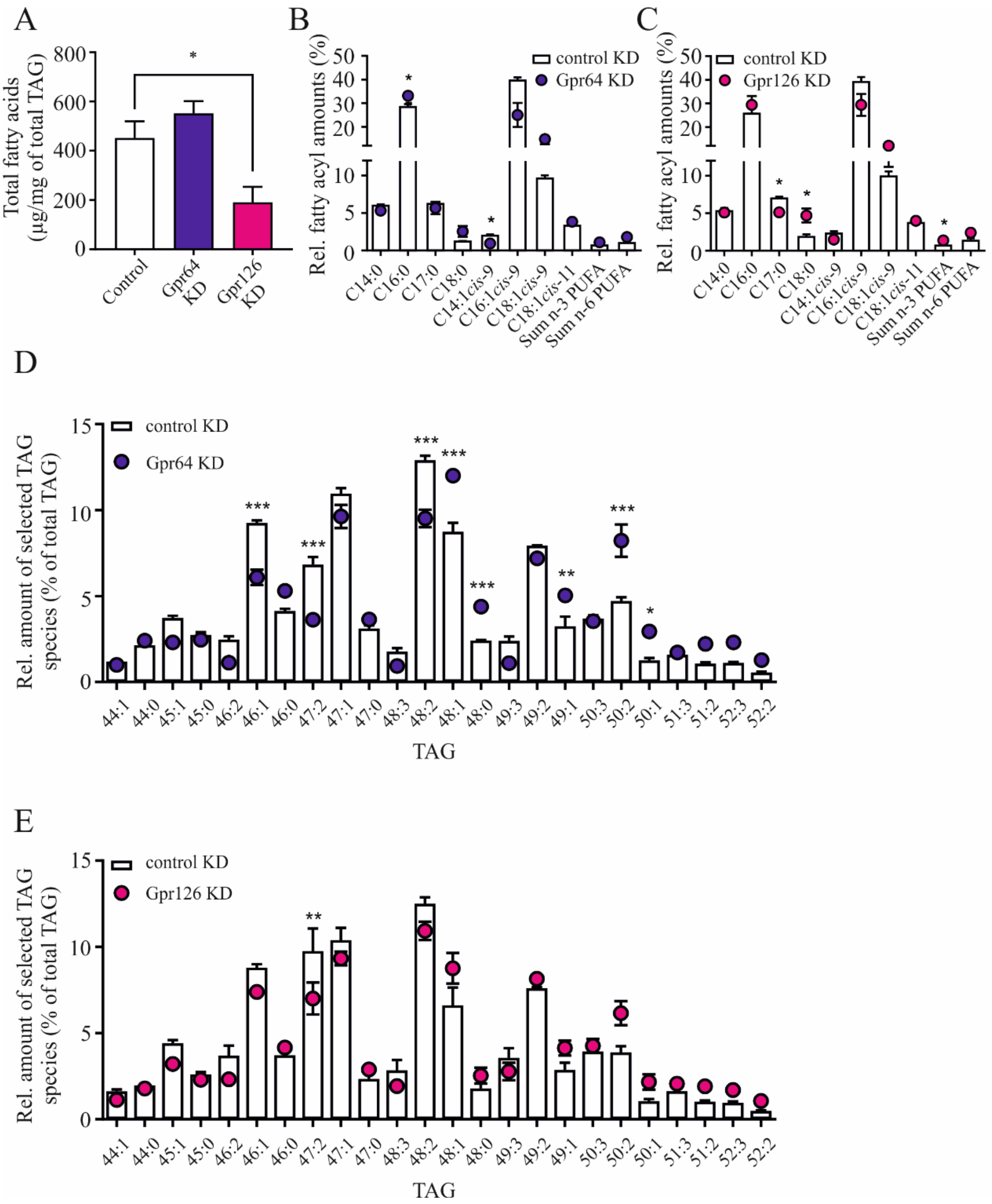

Suppl. Fig. S3. Acyl compositions of triacylglycerols (TAG) in Gpr64 and Gpr126 knock-down 3T3-L1 cells. (A) Total amount of fatty acids per g of lipid per sample is significantly lower under Gpr126 knock-down but not Gpr64 knock-down. (B and C) Detailed analysis of the fatty acid composition was performed by GC-FID for Gpr64 knock-down (B) and Gpr126 knock-down (C) in 3T3-L1 cells and compared to control siRNA transfected cells. Given is the mean ± SEM (n = 3 biological replicates). Significance was assessed by multiple t-test. *p < 0.05. (D and E) The acyl composition was assessed by ESI-MS in day 10-differentiated adipocytes under transient knock-down of Gpr64 (A) and Gpr126 (B) compared to control siRNA-transfected cells. Given is the mean ± SEM (n = 3 biological replicates). Significance was assessed by 2-way ANOVA followed by Dunnett's test for multiple comparisons. *p < 0.05; **p < 0.01; ***p < 0.001, ****p < 0.0001.

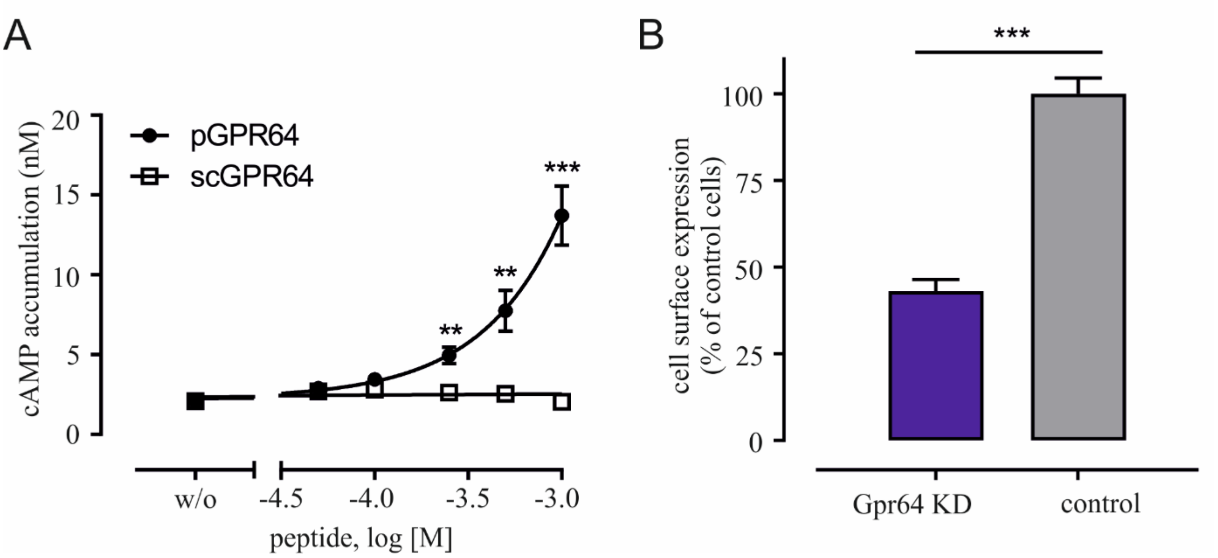

Suppl. Fig. S4. GPR64 function in mature adipocytes. (A) Endogenous GPR64 in 3T3-L1 cells can be activated concentration-dependently with a synthetic peptide (pGPR64) derived from the tethered agonist sequence. A scrambled version of the peptide (scGPR64) used for control purposes does not induce cAMP formation. Shown is the mean ± SEM of 6 experiments each performed in triplicates. (B) siRNA mediated knock-down of Gpr64 results in a significantly reduced cell surface expression of the receptor. Given is the mean ± SEM of 3 independent experiments each performed in triplicates. Cell surface expression of GPR64 following siRNA-mediated knock-down of the receptor is given as percentage of control-transfected 3T3-L1 cells (OD values: 0.21 ± 0.02). Statistical significance was tested using a 2-tailed unpaired t-test $^{**}p < 0.01$; $^{***}p < 0.001$.
